# The effect of an interactive cycling training on cognitive functioning in older adults with mild dementia: study protocol for a randomized controlled trial

**DOI:** 10.1186/s12877-017-0464-x

**Published:** 2017-03-21

**Authors:** E. G. A. Karssemeijer, W. J. R. Bossers, J. A. Aaronson, R. P. C. Kessels, M. G. M. Olde Rikkert

**Affiliations:** 1 Department of Geriatric Medicine, Donders Institute for Brain Cognition and Behaviour, Radboud university medical center, Nijmegen, The Netherlands; 2 Radboud university medical center, Radboudumc Alzheimer Center, PO 9101 (hp 925), Nijmegen, 6500 HB The Netherlands; 3 University of Groningen, University Medical Center Groningen, Center for Human Movement Sciences, Groningen, The Netherlands; 4 Department of Medical Psychology, Donders Institute for Brain Cognition and Behaviour, Radboud university medical center, Nijmegen, The Netherlands

**Keywords:** Dementia, Aerobic training, Combined cognitive-aerobic training, Interactive cycling, Randomized controlled trial, Cognition, Executive functions

## Abstract

**Background:**

To date there is no cure or an effective disease-modifying drug to treat dementia. Available acetylcholine-esterase inhibiting drugs or memantine only produce small benefits on cognitive and behavioural functioning and their clinical relevance remains controversial. Combined cognitive-aerobic interventions are an appealing alternative or add-on to current pharmacological treatments. The primary aim of this study is to investigate the efficacy of a combined cognitive-aerobic training and a single aerobic training compared to an active control group in older adults with mild dementia. We expect to find a beneficial effect on executive functioning in both training regimes, compared to the control intervention, with the largest effect in the combined cognitive-aerobic group. Secondary, intervention effects on cognitive functioning in other domains, physical functioning, physical activity levels, activities of daily living, frailty and quality of life are studied.

**Methods:**

The design is a single-blind, randomized controlled trial (RCT) with three groups: a combined cognitive-aerobic bicycle training (interactive cycling), a single aerobic bicycle training and a control intervention, which consists of stretching and toning exercises. Older adults with mild dementia follow a 12-week training program consisting of three training sessions of 30–40 min per week. The primary study outcome is objective executive functioning measured with a neuropsychological assessment. Secondary measures are objective cognitive functioning in other domains, physical functioning, physical activity levels, activities of daily living, frailty, mood and quality of life. The three groups are compared at baseline, after 6 and 12 weeks of training, and at 24-week follow-up.

**Discussion:**

This study will provide novel information on the effects of an interactive cycling training on executive function in older adults with mild dementia. Furthermore, since this study has both a combined cognitive-aerobic training and a single aerobic training group the effectiveness of the different components of the intervention can be identified. The results of this study may be used for physical and mental activity recommendations in older adults with dementia.

**Trial registration:**

The Netherlands National Trial Register NTR5581. Registered 14 February 2016.

**Electronic supplementary material:**

The online version of this article (doi:10.1186/s12877-017-0464-x) contains supplementary material, which is available to authorized users.

## Background

Dementia is a syndrome characterized by progressive cognitive decline, motor deficits and/or behavioural problems, which increasingly affect the ability to perform activities of daily living [1]. Alzheimer’s disease (AD) is the most common cause of dementia accounting for approximately 60-80% of the dementia cases, followed by vascular dementia [1]. Older age is the strongest risk factor for dementia and due to the aging population the prevalence of dementia is increasing [2]. There are over 9.9 million new cases of dementia each year and the number of persons with dementia is expected to reach 131.50 million in 2050 [3]. Currently, there is no cure or an effective disease-modifying drug to treat dementia [4]. Pharmacological treatment for AD and vascular dementia with acetylcholinesterase inhibitors (rivastigmine, galantamine, donepezil) or memantine produce small benefits on cognitive and behavioural functioning [5, 6]. However, the clinical relevance of these pharmacological treatments is controversial and these drugs can cause adverse events (e.g., anorexia, gastrointestinal problems, insomnia) in this vulnerable patient group [6]. Therefore, we should focus on the development and implementation of non-pharmacological interventions as an alternative or add-on therapy. Physical activity seems to be an appealing option [7, 8], as increased lifetime engagement in physical activity reduces the risk of dementia [9] and recent research shows that older adults with dementia spend approximately two-third of the day being sedentary [10].

Recent meta-analyses show positive effects of aerobic exercise interventions on cognitive function in cognitively healthy older adults, with the largest gains in executive-control processes [11–14]. Executive function refers to higher-order cognitive processes that controls basic, underlying cognitive functions for non-routine, purposeful, goal-directed behaviour and is linked to prefrontal-parietal network activity [15]. Several mechanisms have been identified that may explain this beneficial effect of aerobic exercise on cognitive function: (1) aerobic exercise in aging individuals may increase brain volume, in both grey and white matter, primarily located in the prefrontal and temporal cortices. These brain regions are important for executive control processes and episodic memory, respectively [16, 17]; (2) aerobic exercise may increase the size of the anterior hippocampus, which may lead to improved memory performance [18]; (3) aerobic exercise may enhance neurogenesis in the dentate gyrus of the hippocampus [19]; (4) aerobic exercise may promote extensive cardiovascular changes in the peripheral and cerebral vasculature, such as enhanced angiogenesis [20], and (5) aerobic exercise promotes cardiovascular fitness and therefore reduces peripheral vascular risk factors [21]. Hence, aerobic exercise may have a positive effect on enhancing brain vitality and engagement in physical activity can reduce the risk of dementia-onset in healthy elderly [9].

Several studies have investigated whether physical activity can slow the rate of cognitive decline in older adults with dementia. The results of these studies are mixed. A recently updated meta-analysis of the Cochrane library [22] did not find evidence that physical activity slows cognitive decline in older adults with dementia. In contrast, a meta-analysis of Groot et al. [23] found a beneficial effect of physical activity on cognitive function. This positive effect was independent of the frequency of the intervention and driven by interventions that included aerobic exercise [23]. The opposing outcomes may be explained by the difference in the included studies. Groot et al. incorporated sixteen trials published up to 2015 in the analysis, while the Cochrane library incorporated nine trials and did not include studies after 2013 [22, 23]. Specifically, the most recent studies, reviewed by Groot et al. [23], showed a beneficial effect of physical activity on cognition [24–26]. Moreover, both meta-analyses discuss the large variability in study population, exercise protocols and outcome measures that can complicate interpretation of the results [22, 23].

Studies suggest that the neural and cognitive benefits, elicited by physical activity, can be further enhanced if exposure occurs in the context of a cognitively challenging environment [27–29]. Experimental animal studies have shown that physical activity and environmental enrichment (a combination of complex inanimate and social stimulation [30]) differently affect hippocampal neurogenesis, with physical activity influencing the proliferation of neural precursor cells and enriched environment exerting a survival promoting effect on newborn neurons [27, 29]. The findings of previous studies on cognitive effects of single physical training versus combined cognitive-physical training in healthy older adults are in favour of a combined intervention [31, 32]. These combined interventions also seem to positively influence cognition in persons with dementia, with significant effects found on executive function, attention and processing speed [33]. These potential benefits of a combined cognitive-physical training need further investigation since the limited number and heterogeneity of the conducted studies [33]. Moreover, there is a lack of comparison with single physical training interventions to identify the effectiveness of the different components of the intervention. Thus, methodologically high-quality combined cognitive-physical training compared with single physical training studies are needed.

Earlier studies indicate that the gene Apolipoprotein E (APOE) may be involved as a moderator in the effects of physical activity on cognition [34, 35]. APOE is a cholesterol carrier that supports lipid transport and is involved in brain injury repair [36]. The APOE gene is polymorphic with three major isoforms: ε2, ε3 and ε4 [37]. Carrying the ε4 allele of APOE is the strongest genetic risk factor for developing AD and carrying the ε2 allele is protective [2, 38]. Approximately 14% of the western population carries the ε4 allele and the estimated prevalence of APOE ε4 genotype amongst patients diagnosed with AD is 50% [36, 39]. The risk of developing vascular dementia is also elevated in APOE ε4 carriers, although to a lesser extent [40]. The moderating role of APOE ε4 in the effect of physical activity on cognition is still unknown. Some epidemiological data suggest that physical activity is more protective in APOE ε4 carriers compared to non-carriers with respect to incidence of dementia [21]; cerebral amyloid deposition [34]; cognitive function [35, 41]; cognitive decline [42] and memory-related brain activation [35]. Other studies, however, suggest that physical activity is related to a lower incidence of dementia and higher level of cognitive functioning in APOE ε4 non-carriers [43, 44]. In light of the ‘exercise-is-medicine’ paradigm, insight in APOE ε4 moderation may be relevant for the identification of people who will benefit most from physical activity and cognitive stimulation.

The proposed study will expand the scarce research on the cognitive effects of combined cognitive-aerobic training and single aerobic training in older adults with dementia. Furthermore, explorative data will be collected and analyzed to study the moderating effect of APOE status on cognitive and physical function effects.

### Objectives and hypothesis

The primary objective is to study the effect of a 12-week combined cognitive-aerobic bicycle training on executive functioning, compared to a single aerobic bicycle training and an active control group (i.e. stretching and toning), in community-dwelling older adults with mild dementia. We hypothesize that both training regimes will have a positive effect on executive function, compared to the control intervention, with the largest effect in the combined cognitive-aerobic group.

Secondary objectives include investigating i) the effect of training on the cognitive domains of episodic memory, working memory and psychomotor speed, ii) the effect of the training regimes on physical functioning; iii) the effect of training on activities of daily living, mood, quality of life and frailty, and iv) whether the cognitive effects of training are modified by APOE ε4 carrier state.

## Methods

### Design

**Fig. 1:**
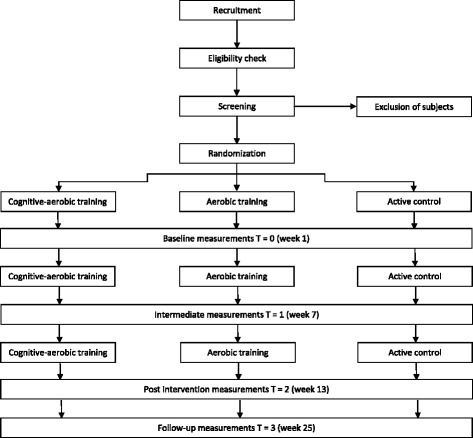
Flowchart of the study design

This study is a single-blind, 12-week randomized controlled trial (RCT) with two experimental intervention groups and one active control group. The study design is illustrated in Fig. 1. Participants will be randomly allocated to one of the intervention groups or the active control group. Primary and secondary outcome measures are assessed at baseline and are repeated after the 12-week intervention period and at 24-weeks in a follow-up assessment. After 6 weeks there is an intermediate measurement consisting of the primary outcome measures.

The study protocol has been approved by the Medical Ethical Committee of Radboud university medical center (Ref No: NL52581.091.15/2015-1857) and is registered at the Dutch trial register (http://www.trialregister.nl) with identification number NTR5581. The study is conducted in compliance with Declaration of Helsinki ethical standards.

### Patient sample and procedures

This study is conducted in community centers in the area of Nijmegen, the Netherlands. The study includes persons with a dementia diagnosis (vascular or Alzheimer or mixed type) aged 60 years and older. Exclusion criteria are: moderate or severe dementia defined by a Mini-Mental Status Examination (MMSE) score of < 17 [22], incapable to give written consent, comorbidities that limit physical activity (e.g., severe cardiovascular conditions, serious neurological or musculoskeletal problems), diagnosis of a major depression or other psychiatric disorder, drug or alcohol dependency, wheelchair bound and severe hearing or visual problems that cannot be corrected with the use of hearing aids/glasses. Furthermore, participants are excluded if they exercise more than five times per week, at least 30 min at a moderate intensity [45].

Participants are recruited through the memory clinic of the Radboudumc Alzheimer Center. Potential eligible participants are notified by their physician about the study. Additionally, participants are recruited through day care centres for elderly with cognitive disorders, advertisement in the local newspapers, and word of mouth. All participants who are interested receive detailed information about the nature, purpose and duration of the study, as well as possible objections, risks of participation and the possibility of withdrawal. The researcher contacts the participant to provide further information (if needed) and to invite them to participate. Subsequently, a screening visit is planned. Prior to the screening visit, written informed consent is obtained from the participants. During the screening visit, inclusion and exclusion criteria are assessed. If necessary, permission is asked by the researcher to access hospital files to further evaluate the in- and exclusion criteria. The inclusion period started in February 2016.

### Interventions and control condition

The study includes two intervention groups, which are a combined cognitive-aerobic bicycle training (interactive cycling) and a single aerobic bicycle training group, and an active control condition (stretching and toning). All training groups receive 30–40 min of training, three times per week for 12 weeks. The training sessions are individually guided by well-trained research assistants. The trainer records the intensity and duration for each training session. In case of missed training sessions, the reason of absence is recorded. The training sessions are carried out at the participating community centres or day care centres in Nijmegen and the surrounding area.

#### Aerobic training

Aerobic training is performed on a stationary bike (Tunturi Go 50). Table 1 presents the progressions in intensity and duration during the training period, adapted from the American College of Sports Medicine (ACSM) Guidelines for Exercise Testing and Prescription [46]. Exercise intensity is prescribed using percentage of hearth rate reserve (HRR). Participants on medication that attenuates heart rate (e.g. beta-blockers) are prescribed exercise intensity using the Borg Rating of Perceived Exertion (RPE; [47]). The RPE asks participants to rate their subjective feelings of exertion [47]. Heart rate is monitored with the Polar® A300 heart rate monitor. The possibility to increase exercise intensity and duration depends on the individual’s physical ability. Duration, intensity and training load are monitored by a trained research assistant.

**Table 1:** Duration and intensity progression of aerobic training

Week	Duration (min)	Intensity (% HRR)	Intensity (RPE)
1&2	20	50–60	12–15
3&4	25	50–60	12–15
5&6	30	60–70	12–15
7–9	35	60–70	12–15
10–12	40	65–75	12–15

#### Interactive cycling training

The interactive cycling training is a combined cognitive-aerobic bicycle training developed by Fietslabyrint (www.fietslabyrint.nl). Additional file 1 shows the training set-up, in which the home-trainer is connected to a video screen. The aerobic training is identical to the training described above. Additionally, the participants are asked to follow a route through a digital environment presented on the video screen and simultaneously perform cognitive tasks that rely on executive functioning. There are different cognitive training levels and the difficulty of the cognitive tasks increases per level, to ensure that the training remains cognitively challenging. Additional file 2 describes the training tasks in further detail. At the end of each training session participants are provided with feedback on their scores on each task and the scores are registered in a diary. When the participants have a response time of less than 5 s and an error rate of less than 5%, they can proceed to the next level.

#### Stretching and toning active control group

Stretching and toning consists of relaxation and flexibility exercises with the same duration and frequency as the other training regimes. The exercises require minimal muscle strength and aerobic capacity and are easy to perform. The level of social engagement is similar to the intervention groups. In persons with dementia, social engagement may have a positive effect on cognitive function [48]. The stretching and toning group thus controls for this social effect. The flexibility exercises consist of upper and lower body exercises, including head rotation, shoulder rotation, shoulders up-down, arm rotation, arm and shoulder muscle strengthening, wrist rotations, flexion/extension fingers, rotation hip, stretching hip flexors and extensors, stretching knee flexors and extensors.

### Outcome measures

#### Primary outcomes

The primary outcome measure of this study is objective executive function. Executive function is measured by four neuropsychological tasks, i.e. the short form of the Trail Making Test part B (numbers 1 to 7 and letters A to G) [49]; the abbreviated 5-line Stroop Color Word Test [50, 51]; Letter Fluency [52, 53] and Rule Shift Cards Test [54] [see Additional file 3]. The tests are administered by trained research assistants before and after the training phase (T0 and T2) and at follow up (T3). Parallel versions are used for letter fluency to minimize learning effects at the second and third time point. All the tests, except for letter fluency, are also administered after 6 weeks (T1). The obtained scores are converted into z-scores based on the standard deviation and mean of the total sample at baseline. Subsequently an executive composite z-score is calculated by averaging the z-scores.

#### Secondary outcomes

##### Cognitive measurements:

Episodic memory, working memory and psychomotor speed are assessed by neuropsychological assessment. Episodic memory is measured with the Location Learning Test – Revised [55], working memory with the Digit Span subtest (forward and backward condition) from the Wechsler Adult Intelligence Scale – Third Edition (WAIS-III) [56] and the Spatial Span from the Wechsler Memory Scale – Third Edition (WMS-III) [57] and psychomotor speed is assessed using the Trail Making Test part A [49] and the reading and color-naming cards from the abbreviated Stroop Color-Word Test [50]. Additional file 3 describes the cognitive measurements and the scoring methods in more detail. The tests are administered at the same time points as executive function (T0, T2 and T3). A parallel version is used at T2 for the Location Learning Test to minimize learning effects. The obtained scores are converted into z-scores and a composite z-score is calculated for each domain.

##### Physical functioning:

Physical functioning is measured with performance-based tests suitable for older people. Physical fitness is assessed with the Åstrand Bike Test [58], mobility with the Timed Up & Go Test [59] and the 10-m Walk Test [60], strength with the 5-times Chair Stand [61] and Handgrip Strength [62] and balance is measured with the Frailty and Injuries Cooperative Studies of Intervention Techniques Subtest 4 [63]. Additional file 4 describes the physical measurements in detail. The different motor domains, strength, physical fitness, balance and mobility, are assessed before and after the training phase (T0 and T2) and at follow-up (T3). At intermediate measurement (T1), only physical fitness and mobility are assessed.

##### Other secondary outcome measures:

Level of physical activity is assessed objectively using an actigraphy device (Philips Actiwatch 2®) that participants wear for seven consecutive days and subjectively with the Physical Activity Scale for the Elderly [64]. The Older Persons and Informal Caregivers Survey Minimum DataSet (TOPICS-MDS) [65] is administered to assess activities of daily living and mood. Quality of life is measured with the Dementia Quality of Life Instrument [66] and frailty by using the Evaluative Frailty Index for Physical Activity [67]. These outcome measures are assessed at pre-test (T0) and post-test (T2). Additional file 4 describes the measures in detail.

#### Moderator

After inclusion, saliva samples are taken with buccal swabs for APOE genotyping. Buccal samples are stored in −20 °C and analyzed using real-time Polymerase Chain Reaction (PCR) [37]. This results in different APOE gene phenotypes: three homozygous (ε2/ε2, ε3/ε3, ε4/ε4) and three heterozygous (ε2/ε3, ε2/ε4, ε3/ε4) [37].

### Sample size

Sample size is determined using software package G*power [68]. The effect size (ES) is estimated based on a previous study on the cognitive effects of combined cognitive-aerobic training and single aerobic training using a similar intervention [69]. In this study a medium effect size (*d* = 0.50) was found for executive functioning after 3 months of training. Therefore, assuming a power of 0.80, an alpha of 0.05 and an expected drop out of 15%, a medium effect size is detected with a total sample size of 171 participants.

### Randomization, blinding and treatment allocation

Participants are randomized after baseline assessments. The minimization technique [70] is used to minimize imbalance between the different groups for gender, severity of cognitive impairment, level of education, use of medication for AD and training location. Minimization is conducted by an independent statistician. Assessors of cognitive outcome measures are blinded to treatment allocation.

### Statistical analysis

Socio-demographic and clinical characteristics at baseline are presented using descriptive statistics. If group differences are observed at baseline, those variables are included as covariates in further analyses. Alpha is set at 0.05 for all analyses. To assess the effect on the primary and secondary outcome measures, analysis of covariance (ANCOVA) is used with cognitive domain scores on the post-tests/intermediate tests as dependent variables, pre-test scores as covariates and group (interactive cycling, aerobic bicycle training, control) as between subject factor. In an explorative analysis the moderating effect of APOE ε4 is evaluated. All analyses are performed as intention-to-treat analysis, including all participants (irrespective of adherence to intervention). Additionally, analyses are rerun as per-protocol analysis. Missing data are substituted using multiple imputation method. Characteristic variables of the sample and cognitive and physical test scores at the different time points will be included in the imputation model. Each imputed dataset will be analysed, pooled and then reported.

## Discussion

Dementia is highly prevalent among older adults. To date no effective disease modifying treatment exists [4]. Combined cognitive-aerobic training seems to be a promising intervention to slow the rate of dementia related cognitive decline. However, up to now, there is insufficient evidence to support its effectiveness. To the best of our knowledge, our study is the first to evaluate the effect of a combined cognitive-aerobic bicycle training and a single aerobic bicycle training on executive functioning in older adults with mild dementia.

One of the major strengths of this study is the design with three groups. Most previous training studies compared a combined cognitive-aerobic training or a single aerobic training with a control group. This study includes both a combined cognitive-aerobic training and a single aerobic training. This gives us the opportunity to assess the differential effects between both training conditions and therewith identify the effectiveness of the different components of the intervention. Another strength is that the difficulty level of the cognitive component in the combined cognitive-aerobic training is adapted to the performance level of the participant. This insures that the training remains cognitively challenging.

A limitation of this study is the relatively short duration of the trial. Previous randomized controlled trials showing cognitive benefits of physical activity or combined cognitive-physical training in older adults with dementia, had intervention periods of 12 weeks or more [7, 33]. We chose an intervention period of 12 weeks to increase the feasibility and adherence rate and minimize drop-out. Another limitation of this study is that the research population is very heterogeneous as older adults with different types of dementia (Alzheimer, vascular or mixed type) are included. This may affect the internal validity of the study. However, the heterogeneous population will increase the external validity of the results of this study to the community dwelling dementia population.

## Conclusions

The results of this study will provide an important contribution to the existing body of knowledge on combined cognitive-aerobic interventions and single aerobic interventions in older adults with dementia. The results of this study can be important for physical and mental activity recommendations in older adults with dementia.

## Additional files

Additional file 1: Bicycle set-up.

Additional file 2: Description of training levels.

Additional file 3: Neuropsychological tests by cognitive domain.

Additional file 4: Secondary outcome measurements.

## Abbreviations

ACSM: American College of Sports Medicine
AD: Alzheimer’s disease
ANCOVA: Analysis of covariance
APOE: Apolipoprotein E
HR: Heart rate
HRR: Heart rate reserve
MMSE: Mini-mental status examination
PCR: Polymerase Chain Reaction
RCT: Randomised controlled trial
RPE: Rate of perceived exertion
TOPICS-MDS: The Older Persons and Informal Caregivers Survey Minimum DataSet
WAIS: Wechsler Adult Intelligence Scale
WMS: Wechsler Memory Scale
