# Supplementary figures and images for: The effect of an interactive cycling training on cognitive functioning in older adults with mild dementia: study protocol for a randomized controlled trial
Source: BMC Geriatr. 2017 Mar 21;17:73. doi: 10.1186/s12877-017-0464-x (PMC5361710; doi:10.1186/s12877-017-0464-x)

**Additional file 1. Bicycle set-up**

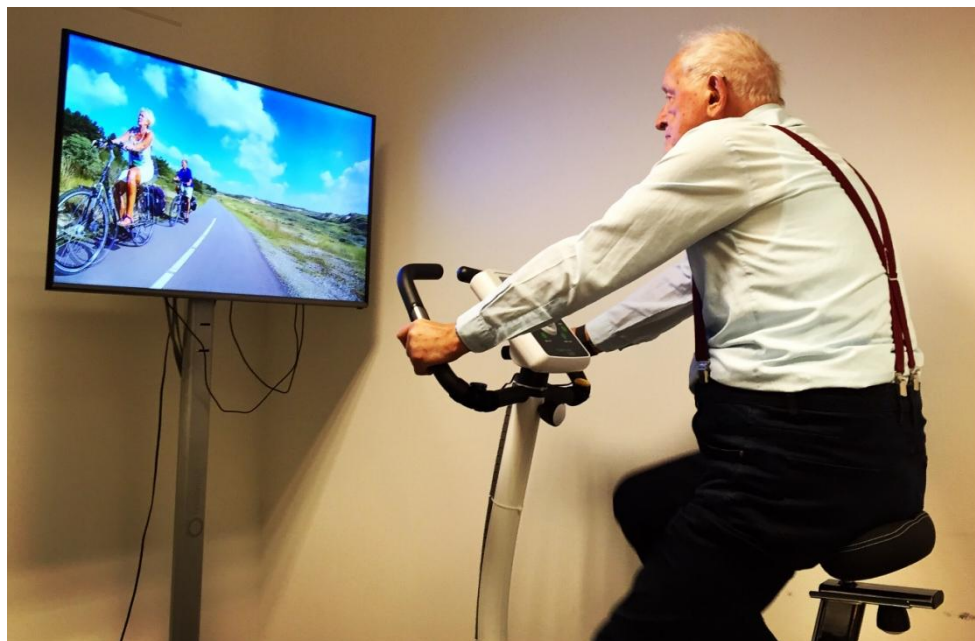

Supplement: Supplementary file 1 — Bicycle set-up. [file 12877_2017_464_MOESM1_ESM.pdf]
