# Supplementary material for: The effect of an interactive cycling training on cognitive functioning in older adults with mild dementia: study protocol for a randomized controlled trial
Source: BMC Geriatr. 2017 Mar 21;17:73. doi: 10.1186/s12877-017-0464-x (PMC5361710; doi:10.1186/s12877-017-0464-x)
Supplement: Supplementary file 2 — Description of training levels. [file 12877_2017_464_MOESM2_ESM.pdf]

**Additional file 2.** Description of training levels

The training software consists of 7 levels, with an increasing difficulty. Each level contains of approximately 15 different cycling routes. An impression of the training software can be found at www.fietslabyrint.nl.

**Level 1**

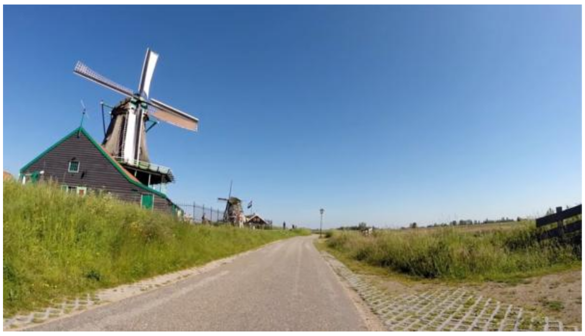

In the first level participants cycle different routes through a digital environment. These routes contain no cognitive tasks.

**Level 2**

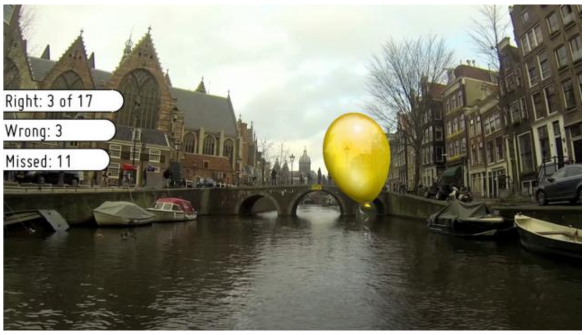


In the second level, balloons (blue, yellow, red) appear on the screen during cycling. When a blue or yellow balloon pops up, participants have to react by pushing on the blue or yellow button, respectively, attached to the steer. When a red balloon pops up participants have to inhibit their response. The balloons are large and remain on screen for ten seconds

**Level 3**

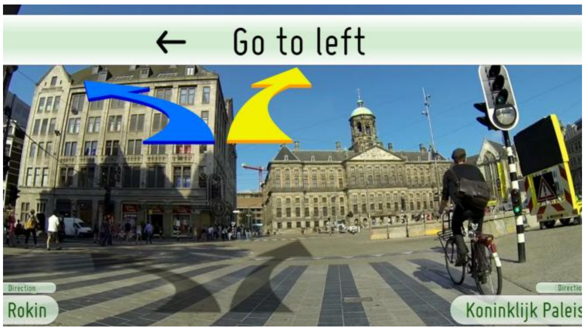


In the third level, participants have to indicate at each junction which direction to go to. This is not voluntarily and will be indicated on the screen by 'go left' or 'go right'.

**Level 4**

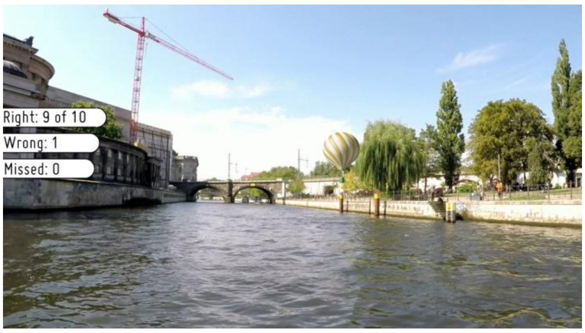


The fourth level is similar to level two. However, the balloons are smaller and stay on screen for only five seconds. Furthermore, air balloons can also appear on the screen. When an air balloon pops up, participants have to inhibit a response.

**Level 5**

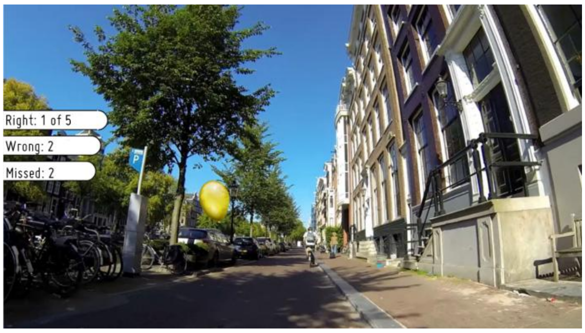


The fifth level is a combination of level three and four. In this level both the balloons, air balloons and directions appear on the screen.

**Level 6**

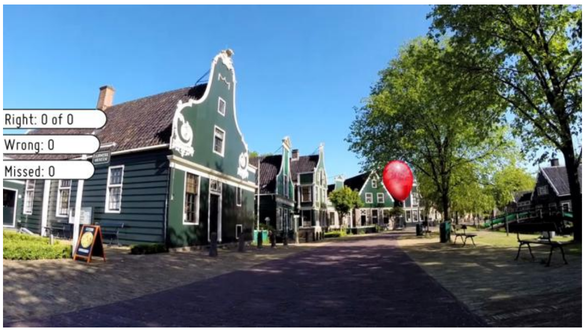


The sixth level is similar to level four; only a set-shifting task element is added. When the participant hears a beep, the task is switched for the next button-press. The participant has to press the blue button if the yellow balloon appears and the yellow button if the blue balloon appears. After pressing the button, the old rules are applicable again.

**Level 7**

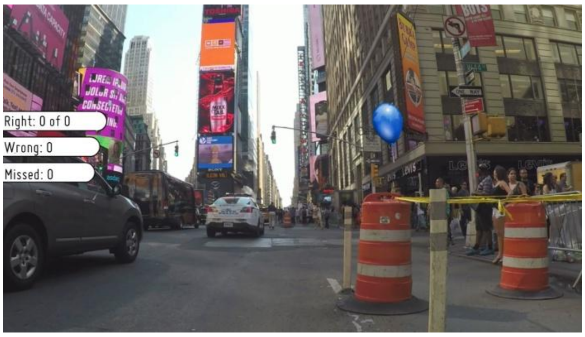


This final level is the most challenging level and contains both directions, balloons, air balloons and the set-shifting task.
