# Supplementary material for: The effect of an interactive cycling training on cognitive functioning in older adults with mild dementia: study protocol for a randomized controlled trial
Source: BMC Geriatr. 2017 Mar 21;17:73. doi: 10.1186/s12877-017-0464-x (PMC5361710; doi:10.1186/s12877-017-0464-x)
Supplement: Supplementary file 3 — Neuropsychological tests by cognitive domain. [file 12877_2017_464_MOESM3_ESM.pdf]

Additional file 3. Neuropsychological tests by cognitive domain

**Executive functioning**
Trail Making Test - part B [49]<br>Participants are asked to connect numbers alternating with letters. Scores are time to completion in seconds. In this study, the short form is administered (numbers 1 to 7 and letters A to G).
Stroop Color-Word Test [50]<br>This test consists of three subtasks: 1) participants are asked to read names of colours (red, green, blue, yellow) printed in black (word-reading card); 2) Participants are asked to name the colour of printed coloured blocks in red, green, blue and yellow (colour-naming card); 3) Participants are presented with coloured words. The ink-colour in which the words are printed needs to be named and the automatic word-reading response needs to be inhibited (colour-word interference card). In this study, the abbreviated version (i.e., the first 5 lines of each card) is administered. Composite scores of accuracy and reaction time are calculated for the colour-word interference card and the colour-naming card. These speed-accuracy trade-off scores are calculated as follows: (100*accuracy) / reaction time [51].
Letter Fluency [52, 53]<br>This test consists of naming words starting with the same letter for 1 minute each. Participants have to name as many words as possible. Scores are the number of correct words. Three equivalent parallel versions will be used: ‘D-A-T’, ‘K-O-M’ and ‘P-G-R’.
Rule Shift Cards Test [54]<br>Participants are asked to respond to a certain rule (part 1; say ‘yes’ if a red playing card is shown, say ‘no’ if a black playing card is presented). In part 2 the rule is changed (say ‘yes’ if the playing card has the same colour as the previous card, say ‘no’ if the colour is different) and participants have to adapt their responses, inhibiting their original response set. Scores are the errors made in part 2.
**Working memory**
Digit Span [56]<br>Sequences of digits (from two to nine digits), are read aloud by the examiner. In the forward condition, participants are instructed to repeat each series in the same order as presented, in the backward conditions, the digits sequences must be reproduced in reverse order. Outcome measure is the number of correctly reproduced items forward and backward. The test is discontinued if participants score zero on both trials of an item.
Spatial Span [57]<br>In this test 9 blocks are fixed on 9 locations on a board. The examiner taps sequences of blocks of increasing length. Participants must repeat the block sequences in the same order (forward condition) or reverse order (backward condition). Outcome measure is the number of correct items forward and backward. The test is discontinued if participants score zero on both trials of an item.
**Memory**
Location Learning Test - Revised [55]<br>The test consists of a 5×5 grid with 10 pictures of easy-to-name everyday objects placed at different locations in the grid. This grid is presented for 15 seconds and participants are subsequently asked to place the pictures on the correct locations in an empty 5×5 grid. Five learning trials are presented, followed by a delayed recall trial after 30 minutes. The total displacement score on the 5 learning trials is the immediate recall score; the displacement score on the delayed trial is the delayed recall score.
**Psychomotor speed**
Trail Making Test – part A [49]<br>Participants are asked to connect numbers in ascending order. Scores are time to completion in

seconds. In this study, the short form is administered (numbers 1 to 14).
Stroop Color-Word Test [50]<br>This test is already described as a measure of executive functioning. Scores on the word-reading and colour-naming cards are measures of psychomotor speed.
