# Supplementary material for: The effect of an interactive cycling training on cognitive functioning in older adults with mild dementia: study protocol for a randomized controlled trial
Source: BMC Geriatr. 2017 Mar 21;17:73. doi: 10.1186/s12877-017-0464-x (PMC5361710; doi:10.1186/s12877-017-0464-x)
Supplement: Supplementary file 4 — Secondary outcome measurements. [file 12877_2017_464_MOESM4_ESM.pdf]

Additional file 4. Secondary outcome measurements

**Physical functioning**
**Physical fitness**
Åstrand Bike Test [58]<br>This is a submaximal exercise test completed according to the Åstrand-Rhyming submaximal protocol. During the first two minutes of the test, resistance of the ergometer is increased until a steady state heart rate (HR) of approximately 70% of the estimated maximal HR is reached. Participants continue cycling for six minutes and each minute HR and RPE is recorded. The maximum oxygen uptake ($V0_{2MAX}$) is estimated using the average HR of minute 5 and 6 and the workload in the Åstrand nomogram.
**Strength**
5-Times Chair Stand [61]<br>Participants are asked to stand-up and sit-down from a chair for five times. The test is performed two times and the fastest time to perform 5 repetitions is reported as a score.
Handgrip strength [62]<br>Handgrip strength is measured using a hand held dynamometer. The dynamometer is set to read force in kilograms. Handgrip strength is measured three times, both left and right, and the highest score is reported.
**Mobility**
Timed Up & Go test [59]<br>For this test subjects are asked to rise from a chair, walk three meters to an orange cone, walk around it and return in their chair. The test is performed two times and the mean time in seconds is used as a score.
10-meter Walk Test [72]<br>This test measures walking speed. Participants are instructed to walk 10 meters at a comfortable pace in a straight line, passing a line set at 2 and 8 meters. The test is performed two times and the fastest time in seconds between 2 and 8 meters is used as score.
**Balance**
Frailty and Injuries Cooperative Studies of Intervention Techniques [63]<br>This test measures static balance control. Participants are asked to perform four different stances and to hold every stance for 10 seconds. The stances are: (1) parallel stand, (2) semi-tandem, (3) tandem, and (4) single-leg without assistive device. The score ranges from 0 to 5 with a higher score indicating better performance.
**Other outcome measures**
Physical Activity Scale for the Elderly [64]<br>This is a questionnaire to assess level of physical activity. It consists of 12 questions regarding the frequency and duration of leisure activity (e.g. sports, jogging, swimming, strengthening and endurance exercise), household activity and work related activity. The total score is computed by multiplying either the time spent in each activity (hours per week) or participation (i.e. yes/no) in an activity, by empirically derived item weights and then summing overall activities.
The Older Persons and Informal Caregivers Survey Minimum DataSet (TOPICS-MDS) [65]<br>Via this questionnaire data is collected on demographics, morbidity, quality of life, functional limitations, mental health, social functioning and health service utilization.
The Evaluative Frailty Index for Physical Activity [67]<br>This is an instrument to evaluate the effect of physical activity on frailty. The questionnaire consists of 50 items on physical functioning, psychological functioning, social functioning and health.
Dementia Quality of Life Instrument [66]<br>This is a quality of life instrument valid and feasible for patients with mild to moderate dementia. The domains self care, physical functioning, social functioning, mood, memory and orientation are

assessed. Each theme has three response options (‘no problems’, ‘some problems’ or ‘extreme problems’).
